# Neural tracking of subjective value under riskand ambiguity in adolescence

**DOI:** 10.3758/s13415-019-00749-5

**Published:** 2019-10-25

**Authors:** Neeltje E. Blankenstein, Anna C. K. van Duijvenvoorde

**Affiliations:** 1 Department of Developmental and Educational Psychology, Institute of Psychology, Leiden University, Leiden, Netherlands; 2 Leiden Institute for Brain and Cognition, Leiden, Netherlands

**Keywords:** Adolescence, Subjective value, Risk, Ambiguity, fMRI, Parametric

## Abstract

Although many neuroimaging studies on adolescent risk taking have focused on brain activation during outcome valuation, less attention has been paid to the neural correlates of choice valuation. Subjective choice valuation may be particularly influenced by whether a choice presents risk (known probabilities) or ambiguity (unknown probabilities), which has rarely been studied in developmental samples. Therefore, we examined the neural tracking of subjective value during choice under risk and ambiguity in a large sample of adolescents (*N* = 188, 12–22 years). Specifically, we investigated which brain regions tracked subjective value coding under risk and ambiguity. A model-based approach to estimate individuals’ risk and ambiguity attitudes showed prominent variation in individuals’ aversions to risk and ambiguity. Furthermore, participants subjectively experienced the ambiguous options as being riskier than the risky options. Subjective value tracking under risk was coded by activation in ventral striatum and superior parietal cortex. Subjective value tracking under ambiguity was coded by dorsolateral prefrontal cortex (PFC) and superior temporal gyrus activation. Finally, overlapping activation in the dorsomedial PFC was observed for subjective value under both conditions. Overall, this is the first study to chart brain activation patterns for subjective choice valuation under risk and ambiguity in an adolescent sample, which shows that the building blocks for risk and ambiguity processing are already present in early adolescence. Finally, we highlight the potential of combining behavioral modeling with fMRI for investigating choice valuation in adolescence, which may ultimately aid in understanding who takes risks and why.

**Electronic supplementary material:**

The online version of this article (10.3758/s13415-019-00749-5) contains supplementary material, which is available to authorized users.

Adolescence encompasses the developmental phase from childhood to adulthood, and is often described as a period marked by increases in risk-taking tendencies such as reckless driving behavior and heightened levels of substance use (Crone & Dahl, 2012; Somerville, Jones, & Casey, 2010). To date, most research on adolescent risk taking has focused on relating reward processes under different conditions of risk, to task-based or real-life risk-taking behavior, and have observed meaningful relations. For instance, higher levels of real-life risk-taking have been associated with attenuated activation in lateral prefrontal regions during reward outcome processing, following decisions under risk (known probabilities) as well as ambiguity (unknown probabilities; Blankenstein, Schreuders, Peper, Crone, & van Duijvenvoorde, 2018). Surprisingly, fewer studies have focused on choice processes, and the development of choice valuation that may drive risk-taking behavior. In particular, classic economic theories posited that expected value—that is, the product of the magnitude and the probability of the outcome, determines choice behavior, in which a higher objective value should be the more attractive choice. However, individuals’ subjective evaluation of choice options rarely matches the objective expected value (Kahneman & Tversky, 1979). Therefore, subjective, rather than objective, choice valuation may be a more sensitive reflection of individual valuation processes (van den Bos, Bruckner, Nassar, Mata, & Eppinger, 2018). Moreover, subjective valuation of risk and ambiguity have been suggested to be sensitive to developmental change (Blankenstein, Crone, van den Bos, & van Duijvenvoorde, 2016; Tymula et al., 2012; van den Bos & Hertwig, 2017), but also shows large individual variation in adolescence (Blankenstein et al., 2016; Blankenstein et al., 2018). To date, few studies have explicitly focused on the behavioral and neural correlates of subjective, and expected, value tracking in adolescents, nor under conditions of risk and ambiguity. In the present study we therefore set out to investigate the behavioral and neural correlates of subjective value tracking under risk and ambiguity in a large sample of adolescents.

One common decision strategy suggested by influential behavioral economic theories such as expected utility theory, further expanded upon by prospect theory, posits that when an individual is confronted with a decision between two alternatives, they first ascertain the subjective value of each available choice option, and then select the option with the highest subjective value (Kahneman & Tversky, 1979). A comprehensive meta-analysis of 206 studies examined the neural basis of subjective value in adults across a wide range of reward types, both during choice and predominantly during outcome (Bartra, McGuire, & Kable, 2013). This meta-analysis identified the anterior insula, dorsomedial prefrontal cortex (DMPFC), dorsal striatum, and thalamus as key regions that have been found to code *both* positive and negative effects of subjective value on brain activation. That is, some studies have found activation increases in these regions with increasing subjective value, whereas others found activation decreases in these regions with increasing subjective value, although these decreases are particularly observed in the loss domain (Bartra et al., 2013). Conversely, the ventral striatum (VS) and ventromedial prefrontal cortex (VMPFC) have been found to predominantly reflect positive effects of subjective value for different reward types (Bartra et al., 2013; Rangel & Clithero, 2014; Sescousse, Caldú, Segura, & Dreher, 2013).

Few studies have examined the neural signature of choice valuation in adolescents. Studies that focused on expected value coding during choice in children, adolescents, and adults (Barkley-Levenson & Galván, 2014; Van Duijvenvoorde et al., 2015), showed that activation in VS, DMPFC, dorsolateral prefrontal cortex (DLPFC), and parts of the parietal cortex were positively related to increases in expected value. In addition, activation in VS was more pronounced with increasing expected value for adolescents compared with adults, highlighting that adolescents are more sensitive to these increases than adults, even when the adolescents were compared with adults who displayed similar gambling behavior (Barkley-Levenson & Galván, 2014). Importantly, these studies focused only on objective expected value scaling in adolescents. However, studies integrating the subjective evaluation of value are currently lacking and may be important because the expected value of a choice option may not exactly match an individual’s subjective value of the choice at hand (van den Bos et al., 2018).

An important factor that contributes to individuals’ (subjective) choice valuation is whether the choice alternatives reflect explicit risk or ambiguous risk. That is, in situations in which the decision outcomes are uncertain, explicit risk (henceforth referred to as risk) reflects decision environments in which the probabilities are known, whereas ambiguous risk (henceforth referred to as *ambiguity*) reflects decision environments in which the probabilities are unknown (Tversky & Kahneman, 1992). Not only are there considerable individual differences in the levels of risk and ambiguity preferences (ranging from aversion to seeking), they may also vary across development and are differentially related to overt risk-taking levels (Blankenstein et al., 2016; Tymula et al., 2012; van den Bos & Hertwig, 2017). That is, behavior under ambiguity has been associated with real-life risky behavior, whereas behavior under risk has not, suggesting that ambiguity may be more reflective of real-life risks (Blankenstein et al., 2016; Tymula et al., 2012; van den Bos & Hertwig, 2017). On the neural level, deciding under conditions of risk and ambiguity have been found to be coded by different brain regions, particularly when considering individual differences in risk-taking levels under risk and ambiguity, in both adults and adolescents (Blankenstein, Peper, Crone, & van Duijvenvoorde, 2017; Blankenstein et al., 2018). On the other hand, a key study comparing neural coding between risk and ambiguity in adults showed that striatum, MPFC, posterior cingulate cortex, and amygdala positively scaled with increases in subjective value under *both* risk and ambiguity (Levy, Snell, Nelson, Rustichini, & Glimcher, 2010). That is, in this study none of these brain regions conveyed unique information about subjective value under either risk or ambiguity. This suggests that at least in adults, subjective value tracking under risk and ambiguity is similarly represented in the brain, even though behavior under these conditions differs considerably. However, how subjective value scaling under conditions of risk and ambiguity is represented in adolescence, has yet to be examined.

In general, this follow-up study to Blankenstein et al. (2018) investigated subjective value tracking under risk and ambiguity, by combining an fMRI gambling task with separately estimated risk and ambiguity attitudes in a large sample of adolescents (*N* = 188, 12–22 years). The goals of this study were twofold. First, we studied which regions code subjective value under risk and ambiguity. Second, we explored whether there were age effects in subjective value coding. We hypothesized that activation in the VS, VMPFC, and parietal cortex in particular would increase with increasing subjective value (Barkley-Levenson & Galván, 2014; Van Duijvenvoorde et al., 2015). Given the mixed findings on DMPFC and insula, we expected that activation in DMPFC and insula could increase or decrease with increasing subjective value (Barkley-Levenson & Galván, 2014; Bartra et al., 2013). Specifically, to assess whether subjective value coding under risk and ambiguity relied on similar (Levy et al., 2010) or separate neural correlates in adolescence, we tested activation patterns for risk and for ambiguity as well as for overlap between these conditions. Although not necessarily within an adolescent age range, prior studies reported age differences in expected value tracking from adolescence into adulthood (Barkley-Levenson & Galván, 2014; Van Duijvenvoorde et al., 2015). Therefore, we explored the linear and quadratic effects of age on the neural tracking of subjective value.

## Method

### Participants

A total of 214 individuals (109 females, 105 males) between 12 and 22 years old participated in this study. Participants were part of a three-wave longitudinal study (Braintime; see, e.g., Peters & Crone, 2017, and Schreuders et al., 2018). Data from this sample (collected at the third wave) have previously been reported in the cross-sectional study by Blankenstein et al. (2018). In this prior study, 18 participants were excluded because of psychiatric disorders, excessive head motion in the MRI scanner (> 3 mm), loss of data, or too few trials in which the gambling option was chosen in the fMRI task. For the goals of the present study, we excluded ten additional participants because of violations of stochastic dominance in at least 50% of trials of the behavioral task (indicating a limited understanding of the task) and because of extreme outliers in risk attitude (i.e., > 3.5 *SD*s above the mean; in- or exclusion of these participants did not qualitatively affect our main behavioral or neural findings). The final sample therefore included 188 participants (100 female, 88 male, *M*_Age_ = 17.18, *SD*_Age_ = 2.59, range 12.02–22.02 years). An overview of the number of participants across ages is provided in Fig. 2a in the Results. IQ was estimated in the first two waves, fell in the normal range, and did not correlate with age (see also Blankenstein et al., 2018; Peters & Crone, 2017; Schreuders et al., 2018).

The institutional review board of the University Medical Center approved this study. Written informed consent was given by adult participants, and by parents in the case of minors (minors provided written assent). All anatomical scans were cleared by a radiologist. Participants were screened for psychiatric or neurological disorders and MRI contra indications (none were observed).

### Wheel of fortune task

#### fMRI task

**Fig. 1:**
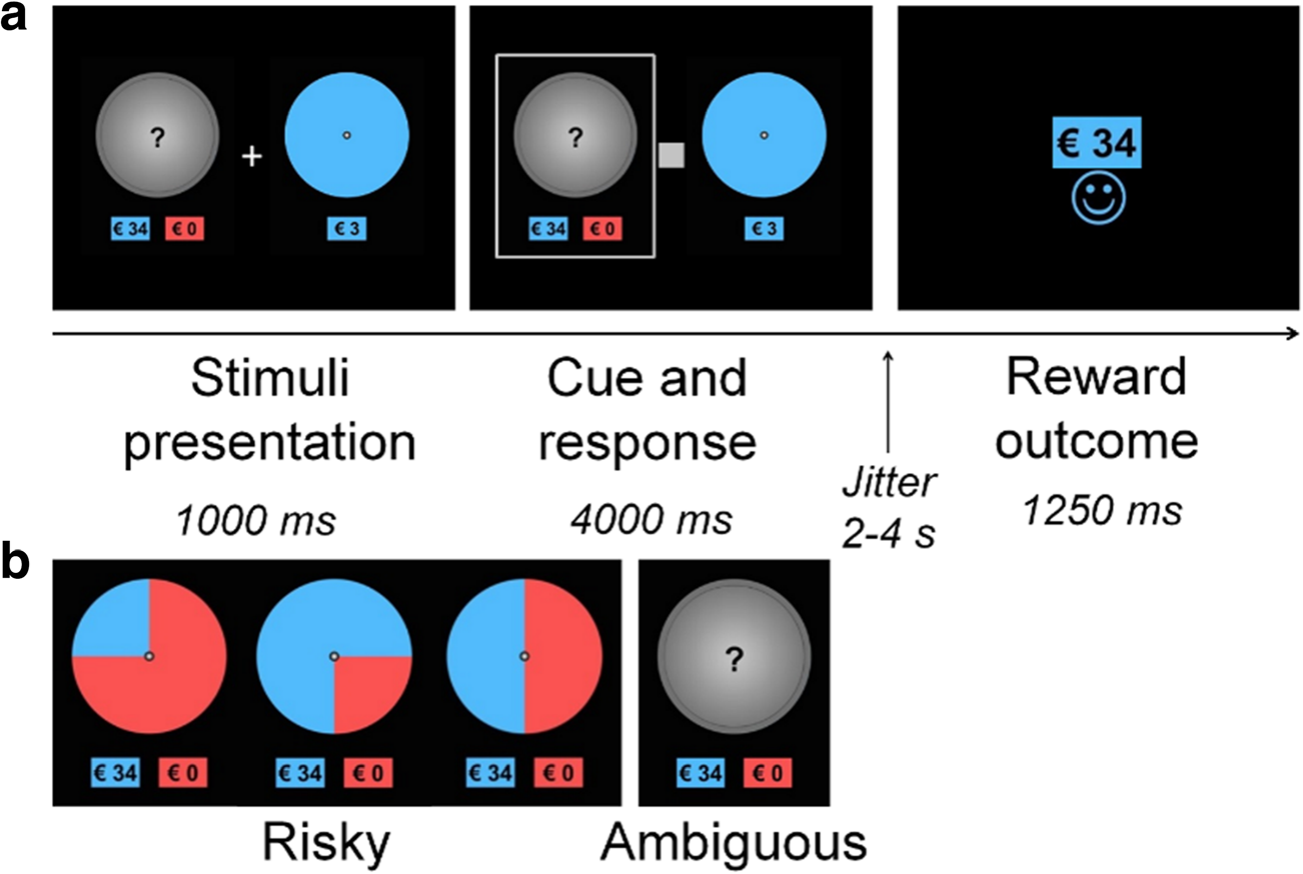
Schematic representation of the fMRI task. **a** Example of an ambiguous trial in which the outcome after choosing to gamble was a gain. **b** Risky and ambiguous stimuli

Participants played a wheel-of-fortune task in the MRI scanner (see Fig. 1; Blankenstein et al., 2017; Blankenstein et al., 2018). Here, participants were asked to make a series of decisions between a “safe” wheel (presenting a consistent sure gain of €3) and a gambling wheel (presenting a chance of winning more money [€31–€34], but also a chance of winning nothing [€0]). The gambling wheel could either be risky (probabilities were known: .25, .50, or .75) or ambiguous (probabilities were hidden). After the decision, participants were presented with the outcome (gain or no gain).

Ninety-two trials were presented: 46 ambiguous and 46 risky trials. Of the risky trials, 30 trials reflected a gamble with a 50% probability of winning, eight trials reflected a gamble with a 75% probability of winning, and eight trials reflected a gamble with a 25% probability of winning. The experiment was programmed such that these probabilities matched the actual probabilities of winning. Furthermore, one of the four possible amounts (€31, €32, €33, or €34) were randomly displayed (without replacement), on a trial-by-trial basis. Thus, although each participant was presented with the same distribution of probabilities, the amount varied per trial.

The task was presented in the scanner via E-Prime (Psychology Software Tools). Participants were presented with the pairs of wheels. Gamble and safe options were randomly displayed on the left or right side of the screen on a trial-by-trial basis, and the position of the blue and red parts of the risky wheels (left, right, bottom, and top of the wheel) were counterbalanced across trials. A gray square prompted the participants to give a response, which had to be given within a 3,000-ms interval. A selection frame around the chosen wheel confirmed the response, and remained visible for the duration of the interval. The decision phase was separated from the outcome phase by a fixation cross of 2–4 s (jittered, with increments of 500 ms). Reward outcomes were presented for 1,250 ms. The intertrial intervals and the optimal trial sequence were determined with OptSeq (Dale, 1999), with jittered intervals varying between 0 and 9,350 ms. In addition, each trial was preceded by a 500-ms fixation cross, which was not part of the intertrial interval.

#### Behavioral task

Following the scan session, participants played a behavioral version of the wheel of fortune task (as validated previously, see Blankenstein et al., 2016; Blankenstein et al., 2017). This task includes more variation in probabilities (.125, .25, .375, .50, .625, .75), amounts (€5, €8, €20, €50), and ambiguity level (0%, 25%, 50%, 75%, 100%), allowing the model-based estimation of each individual’s risk and ambiguity attitudes. No decision outcomes were provided in this task to ensure that the resulting risk and ambiguity attitudes could not be influenced by differences in the choice environment. The task included 24 unique risk trials (all probabilities combined with all amounts), and 16 unique ambiguity trials (all ambiguity levels combined with all amounts). All trial types were presented twice, resulting in 80 trials used for the model-based estimations of risk and ambiguity attitudes.

Each trial started with a jittered fixation cross (500–1,000 ms, with increments of 100 ms) followed by the wheels. A gray square in the center of the screen prompted the participants to respond (reaction time was self-paced), and a selection frame confirmed the participant’s choice. The wheels (gamble, safe) were randomly displayed right and left on the screen, and the position of the blue and red portions of the risky wheels, and the position of the ambiguous lids (top or bottom) were counterbalanced across trials.

### Risk and ambiguity attitude estimations

We estimated each participant’s risk and ambiguity attitude from the behavioral task by modeling the expected utility (EU) of each choice option, using a power utility function with an additional term that takes into account ambiguity attitude as used in previous studies (Blankenstein et al., 2016; Gilboa & Schmeidler, 1989; Levy et al., 2010; Tymula et al., 2012; van den Bos & Hertwig, 2017):

1 $$ EU\left(x,p,A\right)=\left(p-\beta \ast \frac{A}{2}\right)\ast {x}^{\alpha } $$

where *x* indicates the amount, *p* the probability, *A* the ambiguity level, *α* the risk attitude, and *β* the ambiguity attitude. A risk attitude of 1 indicates risk neutrality, a risk attitude of < 1 indicates risk aversion, and a risk attitude > 1 indicates risk-seeking. Relatedly, an ambiguity attitude of 0 indicates ambiguity neutrality (meaning the participant is unaffected by the level of ambiguity), ambiguity attitude > 0 indicates ambiguity aversion (meaning the participants behaves as if the probability is less than the objective probability (50%), and ambiguity attitude < 1 indicates ambiguity seeking (meaning the participant behaves as if the probability is more than the objective probability).For model fitting, the simplex algorithm of the general purpose optimization toolbox (optim) in R was used (R Core Team, 2015). To model trial by trial choices, a logistic choice rule was used to compute the probability of choosing to gamble [Pr(*ChoseGamble*)] as a function of the difference in expected utility of the gamble (*EU*_Gamble_) and the safe option (*EU*_Safe_). Furthermore, the decisions of the participants were modeled as susceptible to an error term (*μ*) to account for potential stochasticity in choice.

2 $$ \Pr (ChoseGamble)=\frac{1}{1+\exp \left(-\left({EU}_{Gamble}-{EU}_{Safe}\right)/\mu \right)} $$

This function was refitted with a grid search procedure to account for local minima in the estimated parameters. To assess how well these attitudes could be detected with these behavioral task settings, we simulated choice behavior (gamble or safe choices) for a range of risk and ambiguity attitudes and ran a parameter recovery analysis. Within the range of most occurring risk and ambiguity attitudes these show reasonably recoveries, see supplementary materials and Fig. S1.

The estimated risk and ambiguity attitudes from our behavioral task were used for behavioral analyses and to set up the parametric regressors for the whole-brain fMRI analyses (see the General Linear Model section below). In the supplementary materials we also report the results of analyses on the raw choice behavior in the behavioral task (Fig. S2). In brief, these results show that participants were sensitive to the task parameters (amount, probability, ambiguity level), and thus that participants had a basic understanding of the behavioral task. Furthermore, on average, participants gambled an equal amount in the risky and ambiguous trials, but responded slower in the ambiguous than in the risky trials.

### Exit questions subjective experience

To examine participants’ subjective experience of the gambling wheels in the behavioral task, we presented participants with a number of exit questions following the behavioral task. Specifically, we presented participants with the different risky (.125, .25, .375, .50, .625, and .75 probabilities) and ambiguous (25%, 50%, 75%, 100%) wheels, without showing the amounts, and asked participants for each of these wheels how risky they found this wheel. Participants could indicate their perceived riskiness on a slider bar (0–100).

### Procedure

The procedure was similar to that of Blankenstein et al. (2017; Blankenstein et al., 2018). Participants were accustomed to the MRI environment using a mock scanner and received instructions on the wheel of fortune task in a quiet laboratory room. We explained participants that the ambiguous wheel could reflect a gamble of any of the risky probabilities (25%, 50%, 75%). In addition, we explained that the computer would select the outcomes of three random trials, of which the average amount was paid out in addition to the standard payout. Eventually, the computer selectively drew a gain, a no gain, and a safe gain outcome (or a gain and two no gain outcomes if the participant never chose the safe option). This draw amounted to an additional rounded payout of €11 or €12 for each individual. Participants completed ten practice trials. In the scanner, participants responded to the task with their right hand using a button box, and head movements were restricted with foam padding. The fMRI task was followed by a high-definition structural scan.

After the MRI session, participants completed the behavioral version of the wheel of fortune task (see also Blankenstein et al., 2017), in which participants were given a hypothetical choice task and were instructed to choose which option they preferred. To explain the different ambiguity levels, we showed the different “lids” that varied in size and covered different proportions of the wheel, and showed the wheels that could lie underneath these lids. Participants practiced three trials beforehand.

Finally, participants completed the exit questions on their subjective experience of the wheels presented in the behavioral task, via Qualtrics (www.qualtrics.com). For other procedural details of the Braintime study that are not related to the current research goals, please see Blankenstein et al. (2018), Schreuders et al. (2018), and Peters and Crone (2017).

### MRI data acquisition

We used a 3-T Philips scanner (Philips Achieva TX) with a standard whole-head coil. Functional scans were acquired during two runs of 246 dynamics each, using T2* echo-planar imaging (EPI). Volumes covered the entire brain [repetition time (TR) = 2.2 s; echo time (TE) = 30 ms; sequential acquisition, 38 slices; voxel size 2.75 × 2.75 × 2.75 mm; field of view (FOV) = 220 × 220 × 114.68 mm]. To allow for equilibration of T1 saturation effects we discarded the first two volumes. A high-resolution 3-D T1 scan was obtained after the fMRI task for anatomical reference (TR = 9.76 ms, TE = 4.59 ms, 140 slices, voxel size = 0.875 mm, FOV = 224 × 177 × 168 mm).

### MRI data analyses

#### Preprocessing

MRI preprocessing steps were identical to Blankenstein et al. (2018). Data was analyzed using SPM8 (Wellcome Department of Cognitive Neurology, London). Images were corrected for slice timing acquisition and rigid body motion. We spatially normalized functional volumes to T1 templates. Translational movement parameters never exceeded 3 mm (< 1 voxel) in any direction for any participant or scan. The normalization algorithm used a 12-parameter affine transform with a nonlinear transformation involving cosine basis function, and resampled the volumes to 3-mm^3^ voxels. Templates were based on MNI305 stereotaxic space. The functional volumes were spatially smoothed using a 6-mm full width at half maximum (FWHM) isotropic Gaussian kernel.

#### General-linear model

We used the general linear model (GLM) in SPM8 to perform statistical analyses on individual subjects’ data. The fMRI time series were modeled as a series of two events: the decision phase and the outcome phase, convolved with a canonical hemodynamic response function (HRF). The onset of the decision phase was modeled with a duration of the participant’s response (1,000 ms + response time; see Fig. 1), and the onset of the outcome phase (gain or no gain) was modeled with zero duration. Events were modeled separately for risk and ambiguity. The GLM included the direct and parametrically modulated regressors of risk and ambiguity during the decision phase, and the direct regressors of gains and no gains during the outcome phase. In the present study, we were interested in the parametric tracking of subjective value under risk and ambiguity only, but in the supplements we show the main effects of choosing under risk and ambiguity (i.e., not parametrically modulated; Fig. S3). The results of the main contrasts during the choice and outcome phase are reported in Blankenstein et al. (2018).

Subjective value under risk and ambiguity were inferred by entering each individual’s risk and ambiguity attitude, derived from the behavioral task, in Eq. 1 for the trials in the fMRI task. That is, for each participant, we determined the subjective value of the wheel chosen by the participant (gamble or safe), given the probability (.25, .50, .75, or 1), amount (€3, €31, €32, €33, or €34), and ambiguity level (0 or 1) of the selected wheel, and the participant’s risk and ambiguity attitude derived from the behavioral task. For instance, an individual who chose the gambling option when presented with an ambiguous gamble of €33, given an ambiguity attitude of *β* = – .318 and risk attitude of *α* = .516, would have a subjective value of this particular trial of 3.99 [i.e., .5 – ((– .318)×(1/2)) × (33^.516) = 3.99]. This was done for each trial and for each individual separately. Parametric values across trials were demeaned per participant.

Trials on which participants did not respond were modeled separately as a regressor of no interest, and six motion parameters were included as nuisance regressors. The least-squares parameter estimates of the height of the best-fitting canonical HRF for each condition separately were used in pairwise contrasts. These pairwise comparisons resulted in individual-specific contrast images, which we used for the higher-level group analyses. All higher-level group analyses were conducted with family-wise error (FWE) cluster correction (*p* < .05, using a primary voxel-wise threshold of *p* < .001, uncorrected; Blankenstein et al., 2017; Woo, Krishnan, & Wager, 2014). We used the MarsBaR toolbox (Brett, Anton, Valabregue, & Poline, 2002; http://marsbar.sourceforge.net) to visualize the patterns of activation, in clusters identified in the whole-brain results. Coordinates of local maxima are reported in MNI space.

## Results

### Behavioral results

#### Model-based risk and ambiguity attitude

**Fig. 2:**
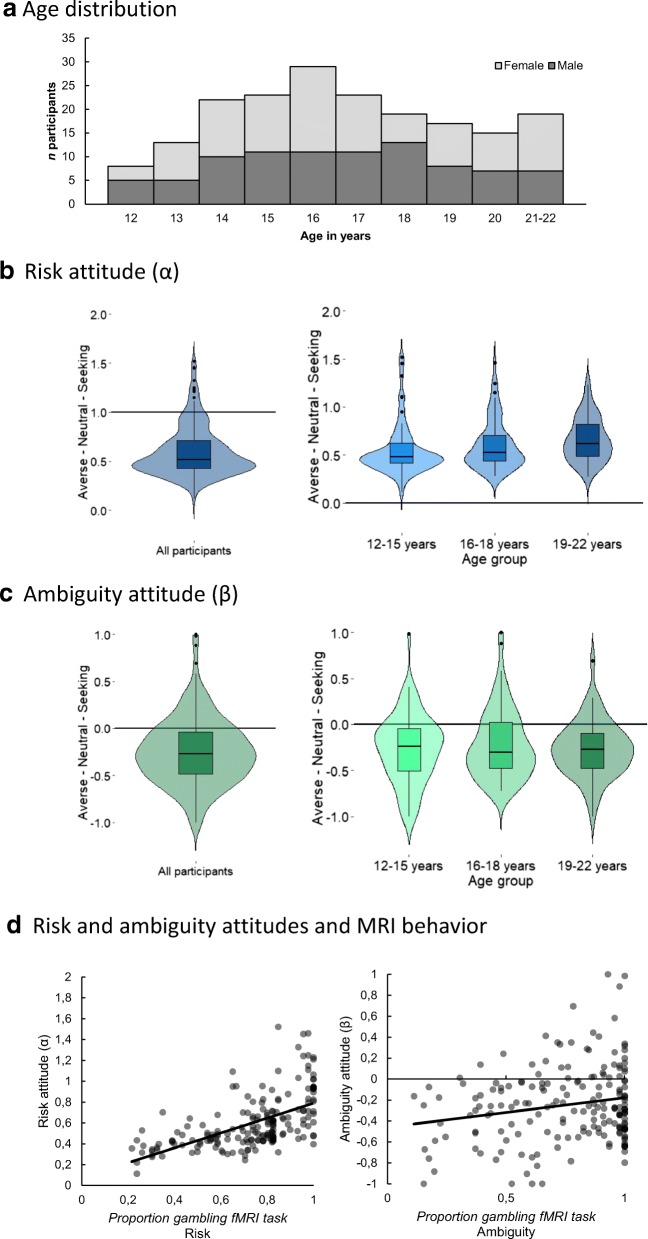
**a** Participant distribution across ages. **b** Violin- and box-plots for risk attitude for all participants and for three age bins. The violin plots show the full distribution of the data. 0 indicates *risk aversion*, 1 indicates *risk neutrality*, and 2 indicates *risk seeking*. **c** Violin- and box-plots for ambiguity attitude for all participants and for three age bins. – 1 indicates *ambiguity aversion*, 0 indicates *ambiguity neutrality*, and 1 indicates *ambiguity seeking*. For both risk and ambiguity attitudes, participants were generally averse, although there were considerable individual differences. **d** Relation between risk attitude and gambling under risk in the fMRI task (left) and between ambiguity attitude and gambling under ambiguity in the fMRI task. Greater risk seeking was related to higher levels of gambling under risk and under ambiguity in the fMRI task. Greater ambiguity seeking was related to higher levels gambling under ambiguity in the fMRI task

First, we formally investigated the model-based estimations of risk and ambiguity attitude. To ease interpretation for these behavioral analyses, we recoded ambiguity attitude such that higher values indicate a relatively more seeking attitude. Figure 2b and c depict box plots of risk and ambiguity attitude, with violin plots superimposed, which show the full distribution of the data for the full sample and across three age bins. On average, participants were generally risk and ambiguity averse (*M*_risk_ = .60, *M*_ambig_ = – .25), although there were considerable individual differences in these attitudes (*SD*_risk_ = 0.26, *range*_risk_ = 0.11–1.52; *SD*_ambig_ = 0.36, *range*_ambig_ = – 1.00 to 1.00). Furthermore, participants did not differ in their degrees of aversion to risk and ambiguity (*p* = .62; as indicated by a paired-samples *t* test on *z*-transformed risk and ambiguity attitudes). Next, we tested for linear, quadratic, and cubic effects of age on risk and ambiguity attitudes using regression analyses. For risk attitude, we observed a positive linear effect of age (*R*^2^ = .02), *F*(1, 186) = 4.29, *b* = *.*015, *SE* = .007 *p* = .04, indicating that risk-seekingness increased slightly across adolescence, whereas no effects of age were observed for ambiguity attitude (all *p*s *>* .1). A partial correlation showed that risk and ambiguity attitudes, when controlling for age, were not significantly correlated (partial *r* = – .083, *p* = .26).

#### Risk and ambiguity attitudes and behavior in the MRI task

In the fMRI task, participants gambled a considerable proportion of times in the risky and ambiguous condition (although there were pronounced individual differences in gambling behavior), but these did not differ significantly (*M*_risk_ = .74, *SE*_risk_ = .015; *M*_ambig_ = .76, *SE*_ambig_ = .018, *p* = .13; no effects of age, linear or quadratic, all *p*s *>* .17). Finally, a repeated measures analysis of variance (ANOVA) with age (linear and quadratic) as a covariate showed that, similar to the behavioral task, participants responded significantly slower in the ambiguous as compared with the risky trials (*M*_ambig_ = 641.59 ms, *SE*_ambig_ = 14.26; *M*_risk_ = 597.06 ms, *SE*_risk_ = 12.89), *F*(1, 185) = 8.79, *p* = .003, *η*_p_^2^ = .045 (no effects of age, linear or quadratic, all *p*s *>* .06).

Furthermore, risk and ambiguity attitudes were correlated with gambling behavior in the MRI scanner (see Fig. 2d). That is, greater risk-seeking attitudes were positively related to increased levels of gambling under risk in the fMRI task (*r* = .581, *p* < .001; when controlling for age: partial *r* = .567, *p* < .001), as well as gambling under ambiguity (*r* = .509, *p* < .001; when controlling for proportion gambling in 50:50 risk trials: partial *r* = .284, *p* < .001; when controlling for 50:50 risk trials and age: partial *r* = .285, *p* < .001). Furthermore, greater ambiguity-seeking attitudes were positively correlated with gambling under ambiguity (*r* = .197, *p* = .007; when controlling for proportion gambling in 50:50 risk trials: partial *r* = .234, *p* = .001; when controlling for 50:50 risk trials and age: partial *r* = .233, *p* = .001), but was not correlated with gambling under risk (*r* = – .022, *p* = .75; when controlling for age: partial *r* = – .02, *p* = .79). These findings indicate that behavior in the MRI scanner reflected the separately estimated risk and ambiguity attitudes, even though the fMRI task used only a selection of risk and ambiguity levels presented in the behavioral task, and included a reward outcome component.

#### Risk and ambiguity attitudes and subjective experience

**Fig. 3:**
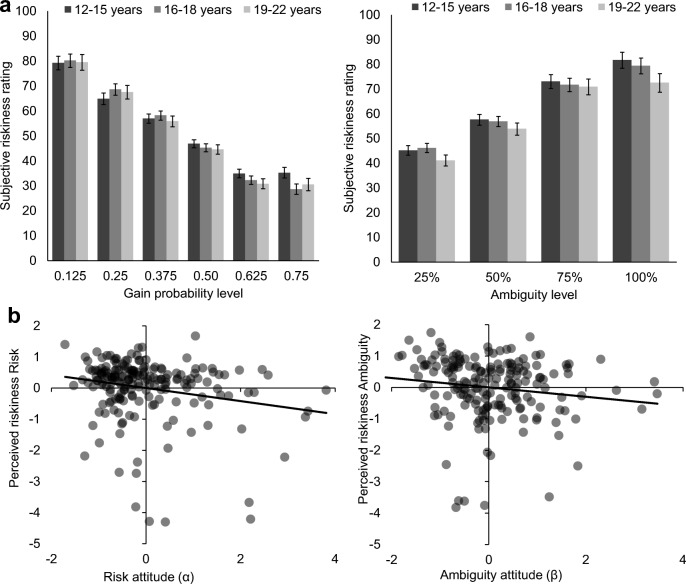
**a** Average perceived riskiness for each of the risky wheels (left) and ambiguous wheels (right) presented in the behavioral task, plotted for three age bins. No age effects were observed. 0 indicates *not all risky*, and 100 indicates *very risky*. Bars indicate standard errors. **b** Partial correlations of risk attitude and the mean perceived riskiness of the risky wheels (left), and partial correlation of ambiguity attitude and the mean perceived riskiness of the ambiguous wheels (right), controlled for age. Risk attitude correlated with the perceived riskiness of both conditions, whereas ambiguity attitude correlated only with the perceived riskiness of ambiguity

To test the robustness of the behavioral estimates of risk and ambiguity attitudes, we examined participants’ responses on the exit questions on perceived riskiness for each of the wheels in the behavioral task. First, a repeated measures ANOVA on the risky wheels with age linear and quadratic as covariates indeed showed that participants subjectively experienced the risky wheels as less risky with increasing gain probability [main effect probability: *F*(5, 920) = 154.99, *p* < .001, *η*_p_^2^ = .457; no effects of age, all *p*s *>* .25]. A similar finding was observed for the ambiguous wheels, in which participants subjectively experienced the ambiguous wheels as more risky with increasing ambiguity level [main effect ambiguity level: *F*(3, 552) = 118.73, *p* < .001, *η*_p_^2^ = .392; no effects of age, all *p*s *>* .14]. On average, participants perceived the ambiguous wheels as being riskier than the risky wheels (*M*_ambig_ = 62.75, *SE*_ambig_ = 1.22, *M*_risk_ = 52.52, *SE*_risk_ = 0.61), *F*(1, 184) = 54.50, *p* < .001, *η*_p_^2^ = .244 (no effects of age, all *p*s *>* .16). Figure 3a depicts the perceived riskiness for each of the probability levels for risk (left) and ambiguity levels for ambiguity (right), plotted for three age bins (12–15 years, 16–18 years, and 19–22 years).

Next we tested whether participants’ average subjective experience was correlated with the behavioral estimations of risk and ambiguity attitude, while controlling for age. These partial correlations showed that risk attitude was negatively correlated with the perceived riskiness of the risky wheels (partial *r* = – .21, *p* = .004), as well as of the ambiguous wheels (partial *r* = – .20, *p* = .007). Thus, a more risk-seeking attitude was correlated with perceiving these wheels as being less risky. Finally, ambiguity attitude was correlated with the perceived riskiness of the ambiguous wheels (partial *r* = – .15, *p* = .043), such that a more ambiguity-seeking attitude was correlated with perceiving these wheels as being less risky. This relation between ambiguity attitude and the perceived riskiness of the risky wheels was not observed (partial *r* = .011, *p* = .88). Together, these findings show that the behavioral estimations of risk and ambiguity attitude also reflect participants’ self-reported subjective experience of the gambles in the behavioral task.

Taken together, these behavioral analyses show that the behavioral task to estimate individuals’ risk and ambiguity attitudes is a valid measure assess subjective valuation in the gambles presented in the fMRI task, and also relates to participants’ self-reported experienced risk in the behavioral task.

### fMRI results

#### Subjective valuations of risk and ambiguity

**Fig. 4:**
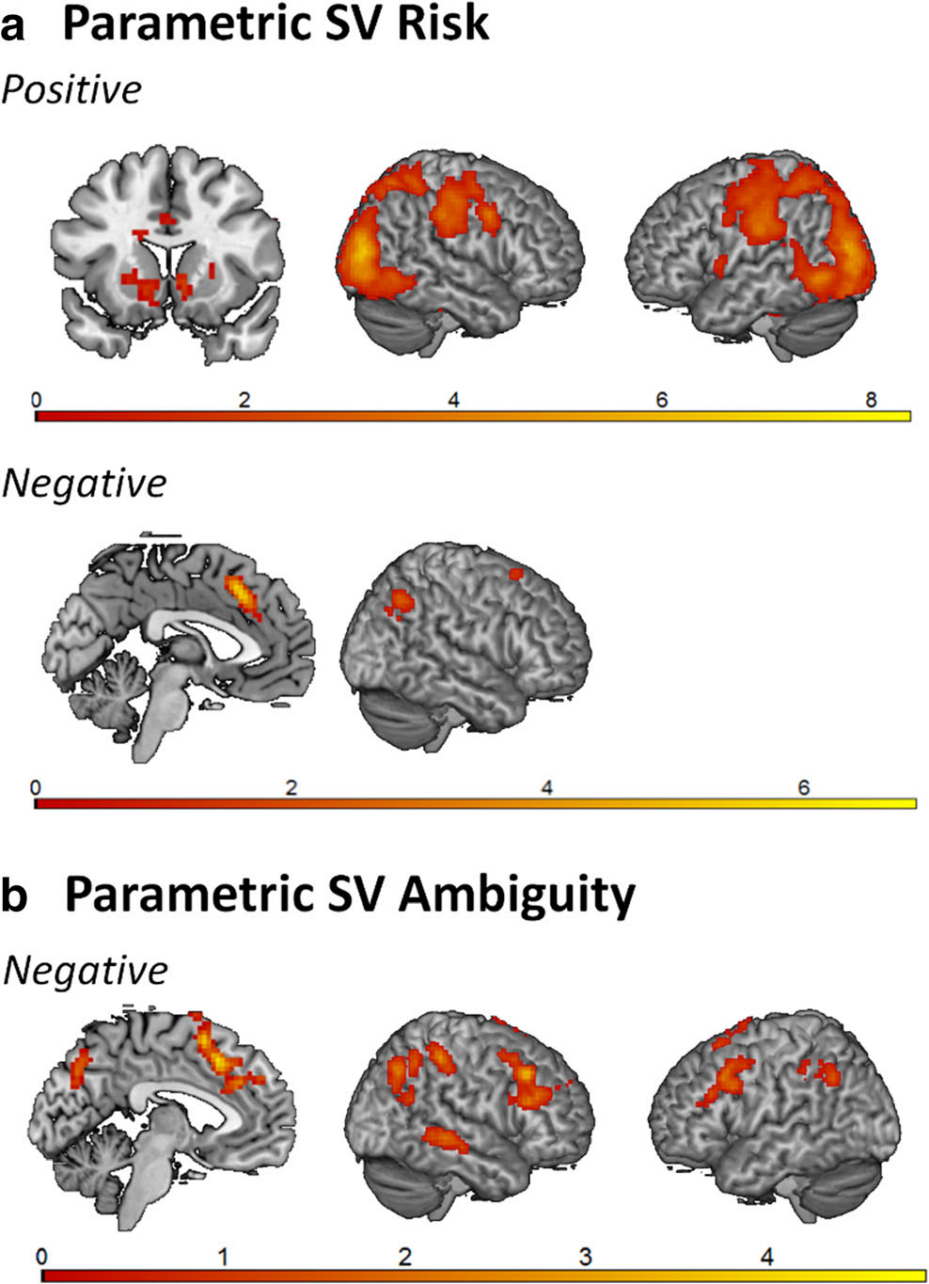
Whole-brain parametric results, showing *t* contrasts. **a** Subjective value (SV) under risk: positive (upper panel; *y* = 14; L; R) and negative (lower panel; *x* = – 4; R). **b** Subjective value under ambiguity: negative (*x* = – 4; R; L). The results are family-wise error cluster-corrected (*p* < .05)

First, we examined the neural patterns of subjective value coding for risk and for ambiguity. To this end, we ran *t* tests of risk and ambiguity (i.e., subjective values per trial for risk and for ambiguity as parametric regressors, see the General-Linear Model section above) against zero and inspected the parametric positive and negative effects. For subjective value under risk, we observed positive parametric patterns of activation in bilateral VS, bilateral superior parietal cortex (SPL), postcentral gyrus, mid-cingulate cortex, and supplementary motor area, indicating that with increasing subjective value, activation in these regions increased. In addition, we observed a negative correlation of activation in DMPFC and right inferior parietal lobe (IPL), indicating decreasing activation with subjective value (Fig. 4a, Table 1).

**Table 1:** Whole-brain results for parametric subjective value (SV) under both risk and ambiguity

Anatomical region		MNI coordinates					
	±	*x*	*y*	*x*	*T*	*k*	*p*
SV risk							
R middle occipital gyrus, including bilat. superior parietal lobe	+	30	– 85	16	8, 36	9,610	< .001
R calcarine gyrus	+	15	– 91	4	7, 83		
R fusiform gyrus	+	30	– 82	– 8	7, 68		
L insula lobe	+	– 33	– 4	16	5, 18	1,028	< .001
L putamen, including R and L caudate nucleus, L inferior frontal gyrus, L thalamus	+	– 30	– 10	– 2	4, 94		
R thalamus	+	21	– 28	– 2	5, 05	299	< .001
R putamen, including R insula lobe	+	27	– 10	7	4, 92		
R middle cingulate cortex, including L middle cingulate cortex	+	12	5	43	4, 17	269	< .001
R supplementary motor area	+	9	– 1	58	4, 08		
L supplementary motor area	+	– 6	– 1	52	3, 54		
L superior medial gyrus	–	– 6	23	40	6, 86	324	< .001
R middle cingulate cortex	–	9	26	34	5, 40		
R supplementary motor area, including R anterior cingulate cortex, R superior frontal gyrus	–	15	20	64	4, 18		
SV ambiguity							
R middle frontal gyrus	–	42	26	43	4, 865	346	< .001
R inferior frontal gyrus	–	54	26	25	4, 277		
R inferior frontal gyrus	–	42	32	28	4, 102		
L supplementary motor area	–	– 3	20	46	4, 656	617	< .001
L supplementary motor area, including R supplementary motor area	–	– 3	11	58	4, 484		
R anterior cingulate cortex, including L superior medial gyrus, L and R superior frontal gyrus, L anterior cingulate cortex	–	12	26	25	4, 388		
R inferior parietal lobe	–	54	– 37	55	4, 48	394	< .001
R angular gyrus	–	36	– 67	43	4, 437		
R inferior parietal lobe	–	48	– 55	52	4, 12		
R middle temporal gyrus	–	66	– 31	– 2	4, 247	146	.001
R middle temporal gyrus	–	63	– 43	1	3, 808		
L inferior frontal gyrus	–	– 51	14	34	4, 211	220	< .001
L middle frontal gyrus	–	– 42	20	40	4, 184		
L middle frontal gyrus	–	– 42	14	49	3, 949		
R precuneus	–	6	– 67	40	3, 959	156	.005
L precuneus, including L cuneus	–	– 3	– 73	37	3, 844		
R cuneus, including L precuneus	–	9	– 79	28	3, 2		
L inferior parietal lobe	–	– 48	– 58	43	3, 924	133	.011
L inferior parietal lobe	–	– 45	– 37	40	3, 637		
L inferior parietal lobe, including L angular gyrus	–	– 48	– 40	52	3, 387		

The results were FWE cluster-corrected (*p* < .05). L = left; R = right. Anatomical labels are based on the Automated Anatomical Labeling (AAL) atlas

For subjective value under ambiguity, we observed parametric subjective value coding also in DMPFC, but in addition, in bilateral DLPFC, right superior temporal gyrus (STG), and bilateral IPL (see Fig. 4b, Table 1). In all these regions, activation was related negatively to subjective value. No positive activation patterns for ambiguity were observed.

Finally, in the supplements we also show these results for a model of objective expected value (Fig. S4, Table S1).

#### Overlap and differences risk and ambiguity

**Fig. 5:**
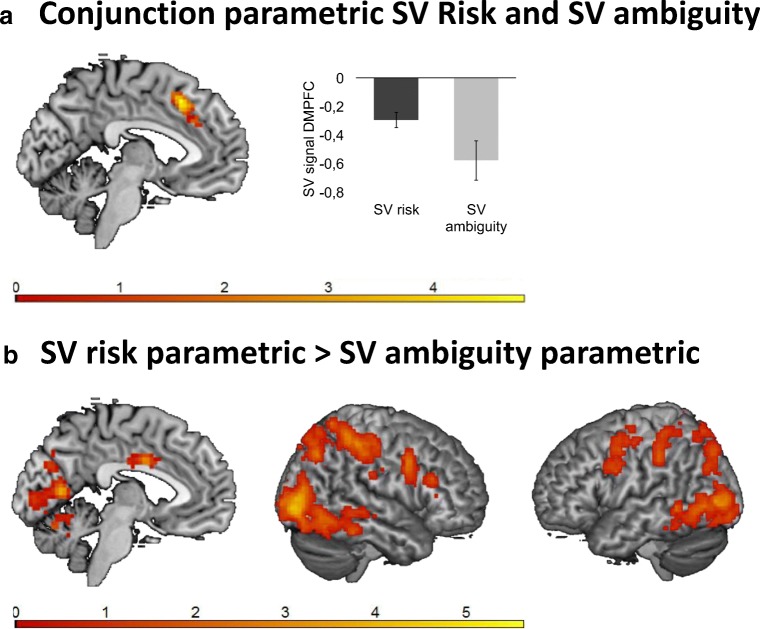
**a** Results of the parametric conjunction between the negative effects of subjective value (SV) under risk and ambiguity (*x* = – 4). The plot on the right is for visualization purposes only. **b** Results of the parametric SV risk > SV ambiguity (*x* = – 4, R; L). The results are FWE cluster-corrected (*p* < .05)

To test for overlap in the patterns of parametric activation during subjective value under risk and under ambiguity, we ran a conjunction analysis on the negative *t* contrasts of risk and ambiguity (no positive activation patterns were observed for subjective value under ambiguity; see above). To this end, we used the “Logical AND” technique, which requires that the contrasts included in the conjunction be individually significant (Nichols, Brett, Andersson, Wager, & Poline, 2005). The conjunction showed significant overlap in the DMPFC for the negative correlations with subjective value under risk and ambiguity (Fig. 5a, Table 2), indicating decreasing activation in this region with subjective value, regardless of condition. Furthermore, contrasting subjective value tracking under risk and ambiguity yielded greater activation in bilateral DLPFC and parietal cortex for subjective value tracking under risk than under ambiguity (see Fig. 5b, Table 2). The reversed contrast did not result in significant findings.

**Table 2:** Results of the parametric conjunction of subjective value (SV) under risk and under ambiguity and of the contrast SV risk > SV ambiguity

Anatomical region		MNI coordinates					
	±	*x*	*y*	*z*	*T*	*k*	*p*
Conjunction SV risk and SV ambiguity							
L supplementary motor area	–	– 3	20	46	4, 66	168	.004
R anterior cingulate cortex	–	9	29	28	3, 86		
L anterior cingulate cortex	–	– 6	32	28	3, 83		
SV risk > SV ambiguity							
R inferior occipital gyrus	+	36	– 91	– 2	5, 63	3,765	< .001
R middle occipital gyrus	+	39	– 85	7	5, 32		
L calcarine gyrus	+	– 9	– 67	7	5, 24		
R superior parietal lobe	+	39	– 46	61	5, 28	899	< .001
R inferior parietal lobe	+	42	– 37	52	4, 79		
R superior occipital gyrus	+	30	– 70	43	4, 75		
L anterior cingulate cortex	+	– 6	2	31	5, 17	101	.035
R middle cingulate cortex	+	6	– 1	31	3, 87		
R anterior cingulate cortex	+	3	8	28	3, 61		
R precentral gyrus	+	54	5	40	4, 75	235	< .001
R inferior frontal gyrus (pars triangularis)	+	54	26	25	4, 46		
R inferior frontal gyrus (pars triangularis)	+	42	29	10	3, 46		
L inferior parietal lobe	+	– 51	– 34	43	4, 44	186	.002
L postcentral gyrus	+	– 48	– 37	61	4, 09		
L inferior parietal lobe	+	– 36	– 43	49	3, 61		
L precentral gyrus	+	– 48	2	31	4, 05	223	< .001
L precentral gyrus	+	– 45	– 1	58	4, 02		

The results were FWE cluster-corrected (*p* < .05). L = left; R = right. Anatomical labels are based on the Automated Anatomical Labeling (AAL) atlas

#### Effects of age

Finally, when including age (linear and quadratic) as a covariate on the *t* contrasts of subjective and expected value under both risk and ambiguity, we observed that these results remained the same, nor did we find any significant effects of age. This indicates that the parametric tracking of subjective and expected value under risk and ambiguity was independent of age in this adolescent sample.

## Discussion

Although many neuroimaging studies on adolescent risk-taking focus on brain activation during outcome valuation, less attention is paid to the neural correlates of choice valuation, especially with regard to risk (known probabilities) and ambiguity (unknown probabilities), which differentially impact real-life risky decision making. This study therefore investigated the neural tracking of subjective value under risk and ambiguity in adolescence, by combining neural activation during an fMRI gambling task with separately estimated risk and ambiguity attitudes. We found activation in bilateral VS and SPL for subjective value under risk and activation in bilateral DLPFC and right STG for subjective value under ambiguity, as well as overlapping activation in the DMPFC for subjective value under both risk and ambiguity. These results were independent of age, and appeared less pronounced when examining expected, rather than subjective, value (reported in the supplementary materials). Finally, behavioral risk and ambiguity attitudes showed limited developmental, but considerable individual indifferences, and echoed participants’ self-reported perceived riskiness of the risky and ambiguous options. The following sections discuss these main findings in further detail.

### Neural tracking of subjective value under risk and ambiguity

On the neural level, we observed that subjective value increases under risk were associated with increased activation in bilateral VS and SPL. Particularly the VS activation coincides with prior adult research on subjective value coding in general (Bartra et al., 2013), and has been suggested to predict risk-seeking choices (Engelmann & Tamir, 2009; Kuhnen & Knutson, 2005; Tobler, O’Doherty, Dolan, & Schultz, 2007). Interestingly, in a previous study we observed that greater risk-seeking attitudes were associated with greater activation in neighboring, valuation, regions (medial and lateral orbitofrontal cortex) during risky gambles (in a separate sample of young adults [18–30 years] using the same experimental paradigms; Blankenstein et al., 2017). The present study extends this earlier work by using a parametric design in which subjective expected value was calculated on a trial-by-trial basis, indicating that neural coding of subjective value under risk is present in a similar set of regions in adolescence. The activation observed in parietal cortex fits well with prior adult research on assessing probabilities (Huettel, Song, & McCarthy, 2005) as well as with risk preference [both functionally (Huettel, Stowe, Gordon, Warner, & Platt, 2006) and structurally (Gilaie-Dotan et al., 2014)]. Finally, in line with these findings, the analyses comparing subjective value under risk with ambiguity showed that PPC coded subjective value increases more pronounced under risk than under ambiguity.

With respect to ambiguity, we observed that activation in superior temporal gyrus and bilateral DLPFC coincided negatively with subjective value. In a separate sample of young adults (18–30 years) we observed that greater ambiguity-seeking attitudes were also associated with heightened superior temporal gyrus activation in a highly overlapping region (MNI coordinates: 63, – 22, – 5; Blankenstein et al., 2017). Thus, the superior temporal gyrus may be presented as a candidate region sensitive to individual differences in ambiguity valuation, although future studies may further investigate its specific direction of activation. Second, DLPFC activation has been suggested to foster exploration tendencies, and thus relates to more ambiguity-seeking attitudes (Huettel et al., 2006). Conversely, the DLPFC has also been associated with heightened cognitive control, and a reduced appetite for risk taking in tasks in which ambiguity can be reduced over time by experience (Fecteau et al., 2007; Knoch et al., 2006). The observation in the present study of decreased DLPFC activation with subjective valuation is in line with this latter interpretation (i.e., a reduced appetite for risk-taking). Together, the STG and DLPFC appear to play a key role in tracking individual differences in subjective value under ambiguity in adolescents.

A conjunction analysis showed that activation in DMPFC coded subjective value both under risk and under ambiguity. The DMPFC (also commonly referred to as dorsal anterior cingulate cortex) has been implicated in a majority of functions related to motivation and cognitive control, including more specifically value comparison and calculation (Hare, Schultz, Camerer, O’Doherty, & Rangel, 2011; Kolling et al., 2016; Piva et al., 2019) and reflections of the expected value of control (Shenhav, Cohen, & Botvinick, 2016). Tentatively, the DMPFC activation observed in the present study may function as a general signal of subjective value coding under different types of uncertainty, given that this region showed overlapping activation for both risk and ambiguity. However, whether this region represents a true neural common currency will need to be established by formal tests such as cross-condition classification (McNamee, Rangel, & O’Doherty, 2013; Kahnt, Park, Haynes, & Tobler, 2014).

Furthermore, in this adolescent study DMPFC negatively coded subjective value in a gain domain, whereas prior literature with adults has shown that negative coding of subjective value occurs predominantly in the loss domain (Bartra et al., 2013). However, the pattern of activation highly overlaps with a recent study with adults that observed reduced DMPFC activation for relative subjective value (i.e., the difference in subjective value between the chosen and unchosen option), using both an intertemporal choice task and a risky mixed gambles task (Piva et al., 2019). Speculatively, given the role of the DMPFC in uncertainty and risk avoidance in adolescents (e.g., Van Duijvenvoorde et al., 2015; Van Leijenhorst et al., 2010) and adults (Xue et al., 2008), the negative value signal in the DMPFC observed in the present study may possibly convey increased aversion to risk and ambiguity. Nonetheless, future studies need to replicate this finding, for instance by including a loss domain, as well as including adult participants.

Some of our findings do not resonate with general prior work on subjective value in adults, given the absence of pronounced positive subjective value encoding during the choice phase in regions such as VS and VMPFC (see, e.g., Bartra et al., 2013), in particular for the ambiguous condition. Our findings also deviate from more similar prior work on subjective value coding under risk and ambiguity specifically, as reported by Levy et al. (2010), who observed that the striatum and MPFC were activated with increasing subjective value under risk as well as under ambiguity. Here we outline a number of reasons for this dissimilarity. First, this may be due to the separate behavioral task we used to estimate risk and ambiguity attitudes, however this seems unlikely because risk and ambiguity attitudes were positively and significantly correlated to fMRI choice behavior. Second, this discrepancy may be due to the fact that we studied a developmental population. However, we did not observe pronounced age effects in our variables of interest, suggesting the building blocks of risk and ambiguity processing are already in place at an early age. It is therefore unlikely that developmental changes underlie the absence of positive subjective value coding for ambiguity. Third, these discrepancies may be due to differences in task designs. As compared to Levy et al., the present fMRI paradigm included a more limited set of probability levels and amounts, and included an outcome phase. In addition, the Levy et al. study included more levels of ambiguity, in which we included one level of complete ambiguity (which was not included in the Levy et al. study). A final possibility for these discrepant findings is that participants may have developed choice heuristics (mental shortcuts) while performing the fMRI task. That is, individuals often use a simple heuristic that maximizes the overall probability of winning (Van Duijvenvoorde et al., 2016; Venkatraman, Payne, & Huettel, 2014). Tentatively, the use of heuristics and lack of variation in the fMRI task may explain why positive signals in typical value regions such as the VS and VMPFC were less pronounced, particularly in the ambiguity condition. Nonetheless, and bearing these methodological consideration in mind, given that this the first study to test subjective value coding under risk and ambiguity in a large adolescent sample spanning a broad age range, these findings may offer an important starting point for future (developmental) studies.

### Subjective and expected value coding under risk and ambiguity

In addition to testing subjective value under risk and ambiguity, we explored whether similar findings were observed in a model testing for objective expected value coding under risk and ambiguity (i.e., Probability × Amount, not weighted by individuals’ risk and ambiguity attitude). Overall, we found similar, but less pronounced, results in this model (reported in the supplements). Specifically, similar to the model with subjective value, we found heightened activation in bilateral VS and SPL for increasing expected value under risk, and in DMPFC and right PPC for decreasing expected value under risk. For ambiguity, only bilateral DLPFC and right IPL with decreasing expected value was observed. Furthermore, we did not observe the overlapping neural coding in DMPFC for decreases in subjective value under risk and ambiguity in the model of expected value. On the one hand, these less pronounced findings may have resulted in less variation in task parameters, and thus to fewer neural changes that could be detected. On the other hand, these findings may suggest that making use of subjective, rather than expected, valuation, is more meaningful when studying the neural underpinnings of (adolescent) choice valuation (Glimcher & Rustichini, 2004; van den Bos et al., 2018), and highlights the potential of this particular method. Nevertheless, formal tests and future studies should replicate our findings, preferably by using a more elaborate task design.

### Effects of age and individual differences in subjective valuation of risk and ambiguity

To assess individuals’ preference toward risk and ambiguity, we made use of a behavioral task and a model-based approach. Concurring with previous findings, we observed that participants were generally risk- and ambiguity-averse, and responded slower in ambiguity compared with risk (Blankenstein et al., 2017). Furthermore, participants subjectively experienced the ambiguous wheels in the task as more risky, compared with the risky wheels. Moreover, we showed that behavioral risk aversion was associated with perceiving the risky and ambiguous wheels in the task as riskier, and ambiguity aversion with perceiving the ambiguous wheels as riskier. This latter finding in particular suggest that these model-based measures not only reflect behavioral tendencies under risk and ambiguity, but also reflect the subjective experience of gambling behavior. In sum, these data suggest meaningful differences between individuals in subjective evaluation of risk under known (risk) and unknown (ambiguity) contexts. This inter-individual variability set the stage for testing our hypotheses on the neural tracking of subjective valuation under risk and ambiguity.

Behaviorally, we observed that risk-seeking slightly increased across adolescence, whereas no developmental change was observed for ambiguity attitudes. Previous findings observed heightened ambiguity tolerance in adolescents compared with adults (Blankenstein et al., 2016; Tymula et al., 2012) and for adolescents as compared with both children and adults (although in a loss frame only; van den Bos & Hertwig, 2017). Furthermore, risk attitudes have been found to either show no developmental trend (Blankenstein et al., 2016), show a quadratic peak in risk seeking in mid adolescents (van den Bos & Hertwig, 2017) or heightened risk aversion in adolescents compared with adults (Tymula et al., 2012). These previous studies included age ranges well into adulthood, or started in early childhood (Tymula et al.: 12–17 years and 30–50 years; van den Bos & Hertwig: 8–22 years; Blankenstein et al., 2016: 10–25 years). Together, the present findings indicate that a developmental window across adolescence and into young adulthood is suitable to test individual variation, but less meaningful to detect developmental change. An interesting next step would be to include young children (< 8 years) and older adults (> 25 years), to establish developmental differences in ambiguity and risk attitudes. Nonetheless, these findings show that risk and ambiguity aversion are already present in early adolescence.

Similar to the behavioral results, we did not observe any age effects (linear, nor quadratic) on neural patterns of activation. Prior studies have observed age differences in the neural tracking of expected value, specifically in VS (more pronounced in adolescents (13–17 years) compared with adults (25–30 years); Barkley-Levenson & Galván, 2014), and in VMPFC and parietal cortex (linear increases from childhood (8–11 years) to adolescence (16–19 years) to adulthood (25–34 years; Van Duijvenvoorde et al., 2015). However, in our previous study including the same participants, few age effects were observed on risk and ambiguity processing during gambling (Blankenstein et al., 2018). Furthermore, the fact that minimal age effects were observed behaviorally in the present study may further explain the absence of age effects on the neural coding of subjective and expected value. Again, including young children and older adults may prove valuable for future studies. Importantly, the present findings indicate that the building blocks for processing risk and ambiguity are already present in early adolescence.

Finally, an interesting avenue for future research is to include loss gambles. The present study shows how adolescents experienced risk and ambiguity, and their corresponding neural patterns, in a gain domain only. Behaviorally, van den Bos and Hertwig (2017) observed differential developmental patterns for risk and ambiguity attitudes under gain and loss contexts, and differential relations with real-life risk-taking behavior. Tentatively, these disparate effects for gain and loss under risk and ambiguity suggest that neural patterns of subjective value under risk and ambiguity in a loss domain may result in different findings as well. For instance, a recent study on gain and loss magnitude tracking in adolescence (13 to 20 years; Insel & Somerville, 2018) observed that gain magnitude tracking was elevated in the striatum during early adolescence, which then gradually decreased across age. However, loss magnitude tracking in the anterior insula followed a quadratic pattern, with lowest activation patterns in mid-to-late adolescence. Although this study focused on objective gain and loss tracking during outcome, this study stimulates hypotheses on subjective value tracking during choice in a loss domain, proposing that the anterior insula is a key region coding losses that may be least pronounced in mid-adolescence. Given the quadratic peak in ambiguity seeking in mid-adolescence under loss as observed by van den Bos and Hertwig, an interesting avenue for future research is to study whether attenuated loss processing in anterior insula in mid-adolescence relates to heightened ambiguity seeking under losses in mid-adolescence, and to what degree this predicts real-world risk-taking behavior.

## Conclusion

In this study, we aimed to extend previous research by explicitly investigating subjective value tracking under risk (known probabilities) and ambiguity (unknown probabilities) in a large sample of adolescents. Our findings suggest that the neural coding of subjective value under risk and ambiguity is reflected in both different and overlapping patterns of brain activation in adolescents in regions such as the DLPFC and DMPFC. Moreover, these findings seem to suggest it is valuable to include subjective, rather than objective, measures of choice valuation in neuroimaging studies on adolescent risk taking. Indeed, behavioral estimations of risk and ambiguity preference showed considerable individual variation, which were reflected in individuals’ self-reported perceived riskiness of the risky and ambiguous choice options. Furthermore, the limited age effects observed in the present study highlight the need for studying a wider age range to unravel these developmental differences with more certainty, but also show that the building blocks for subjective value coding under risk and ambiguity are already present in early adolescence and remain stable across adolescence. In addition, these findings illustrate the potential to investigate individual variation in brain and behavior in adolescence. Together, these findings help to gain insights into subjective valuation under risk and ambiguity—two decision contexts that differentially impact real-life risk taking—in adolescents. Finally, this study highlights the potential of combining model-based behavioral analyses with fMRI. Such a mechanistic understanding may ultimately aid in understanding who takes risks and why.

## Electronic supplementary material

ESM 1 (DOCX 1385 kb)
