# Supplementary material for: Neural tracking of subjective value under riskand ambiguity in adolescence
Source: Cogn Affect Behav Neurosci. 2019 Oct 25;19(6):1364–78. doi: 10.3758/s13415-019-00749-5 (PMC6861198; doi:10.3758/s13415-019-00749-5)
Supplement: Supplementary file 1 — (DOCX 1385 kb) [file 13415_2019_749_MOESM1_ESM.docx]

**Supplementary Materials**

***Simulations and parameter recovery***

To test whether risk and ambiguity attitude could be recovered under the current task settings, we simulated choice behavior (gamble or safe choices) for our task settings for a range of risk attitudes (i.e., attitudes ranging from 0 to 2 in steps of 0.2) and for a range of ambiguity attitudes (attitudes ranging from -1 to 1, in steps of 0.2). For each combination for risk attitude and ambiguity attitude we simulated 100 subjects, resulting in a total of 12100 subjects. Next, on these simulated choice data we estimated risk and ambiguity attitudes following the procedure as described in the main text under ‘Risk and ambiguity attitude estimations’. Parameter recovery is shown in Figure S1. We focused specifically on the range of most-occurring risk and ambiguity attitudes in the behavioral data. These plots show that risk-attitudes were recovered, although were slightly underestimated for risk averse, and overestimated for risk seeking subjects in simulated datasets. Similarly, ambiguity attitudes recovered well, although were slightly underestimated for ambiguity-seeking subjects. In our currently collected behavioral data, risk and ambiguity attitudes rarely met these extreme values. Together, these simulations suggest that our behavioral task and modelling procedures seem suitable to estimate risk and ambiguity attitudes.

*Figure S1.* Simulated (x-axes) and recovered (y-axes) risk attitudes and ambiguity attitudes, in box plots. The upper panel (**A-C**) shows recovery for risk attitude, in which higher α’s on the axis indicate more risk seeking (α = 1 indicates risk neutrality). The different plots display recovery of risk attitude for specific levels of ambiguity attitudes that were evident in the real data (left to right displays low to high ambiguity aversion). The lower panel (**D-F**) shows recovery for ambiguity attitude, in which higher β’s indicate more ambiguity aversion (β = 0 indicates ambiguity-neutrality). The different plots display recovery of ambiguity attitude for specific levels of risk attitude that were evident in the real data (left to right displays risk aversion to risk neutrality).

**Choice behavior behavioral task**

To investigate whether participants had a basic sensitivity to the parameters (amount, probability, ambiguity level) of the task outside the scanner, we examined raw choice behavior. Repeated measures ANOVAs with age group (12-16 years and 17-22 years, in line with Blankenstein et al., 2016) as a between-subjects factor showed that gambling behavior increased with increasing probability and amount, and decreased with increasing ambiguity level (see Figure S2; main effect probability: *F*(5, 930) = 570.91, *p* < .001, η^2^ = .754; age group * probability interaction effect: *p* = .498; main effect amount: *F*(3, 558) = 915.59, *p* = .83, η^2^ = .831, age group * amount interaction effect: *F*(3, 558) = 6.65, *p* < .001, η^2^ = .034; main effect ambiguity level: *F*(3, 558) = 48.54, *p* < .001, η^2^ = .207, age group * ambiguity level interaction: *p* = .637). Thus, participants were sensitive to these parameters, indicating a general understanding of the task. Finally, paired-samples *t-*tests showed that on average, participants gambled an equal amount in the risky and ambiguous trials (*p* = .375, *M_risk_* = .35, *SE_risk_* = .007, *M_ambig_ =* .36, *SE_ambig_* = .01), but that participants responded significantly slower in the ambiguous than in the risky trials (*t*(187) = 3.462, *p* = .001, *M_risk_* = 470.75, *SE_risk_* = 18.15, *M_ambig_ =* 495.11, *SE_ambig_* = 18.96).


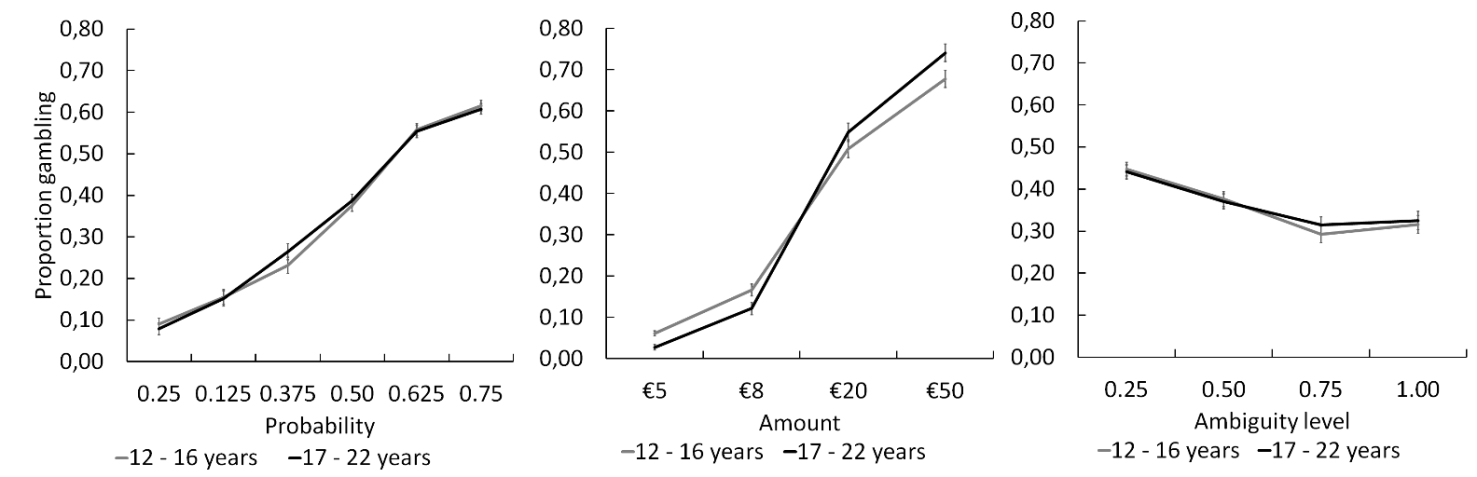


*Figure S2***.** Proportion gambling for probability (left), amount (middle), and ambiguity level (right) per age group.

**Main activation during fMRI task**

**
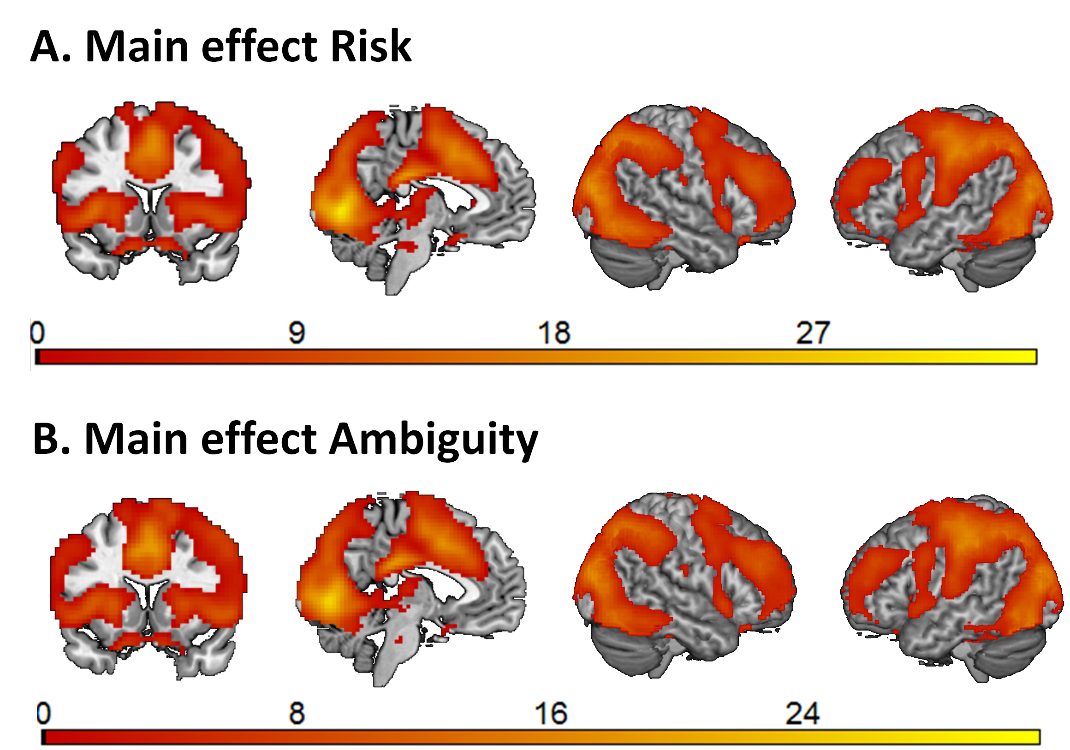
**

*Figure S3.* Activation in the fMRI task during the decision-making phase (not parametrically modulated). **A.** Activation during risky decisions versus fixation (y = 14; x = -4; L; R). **B.** Activation during ambiguous decisions versus fixation (y = 14, x = -4; L; R). Results were FWE voxel-corrected (*p* < .001).

**fMRI results for expected value**

We tested whether the effects of risk and ambiguity, and the conjunction, were also present in a model testing for effects of objective expected value (i.e., product of probability and amount, not weighted by individuals’ risk and ambiguity attitude). To this end we again ran *t*-test on subjective value under risk and under ambiguity (as parametric regressors). First, the positive effects of risk were highly similar in the expected value model, compared with the subjective value model, with activation in bilateral VS, SMA, and SPL, whereas the negative effects showed additional activation in bilateral VLPFC (which was also present in the model with subjective value but did not survive cluster correction; Table S1, Figure S4A). For ambiguity, activation was less pronounced in left DLPFC and right IPL, and absent in DMPFC and left IPL (Table S1; Figure S4B). As a result, in a conjunction analysis we observed that the activation in DMPFC observed for the negative effects of risk and ambiguity in subjective value, was not present in the model with expected value. Finally, as in the model with subjective value, all of these findings were independent of age.


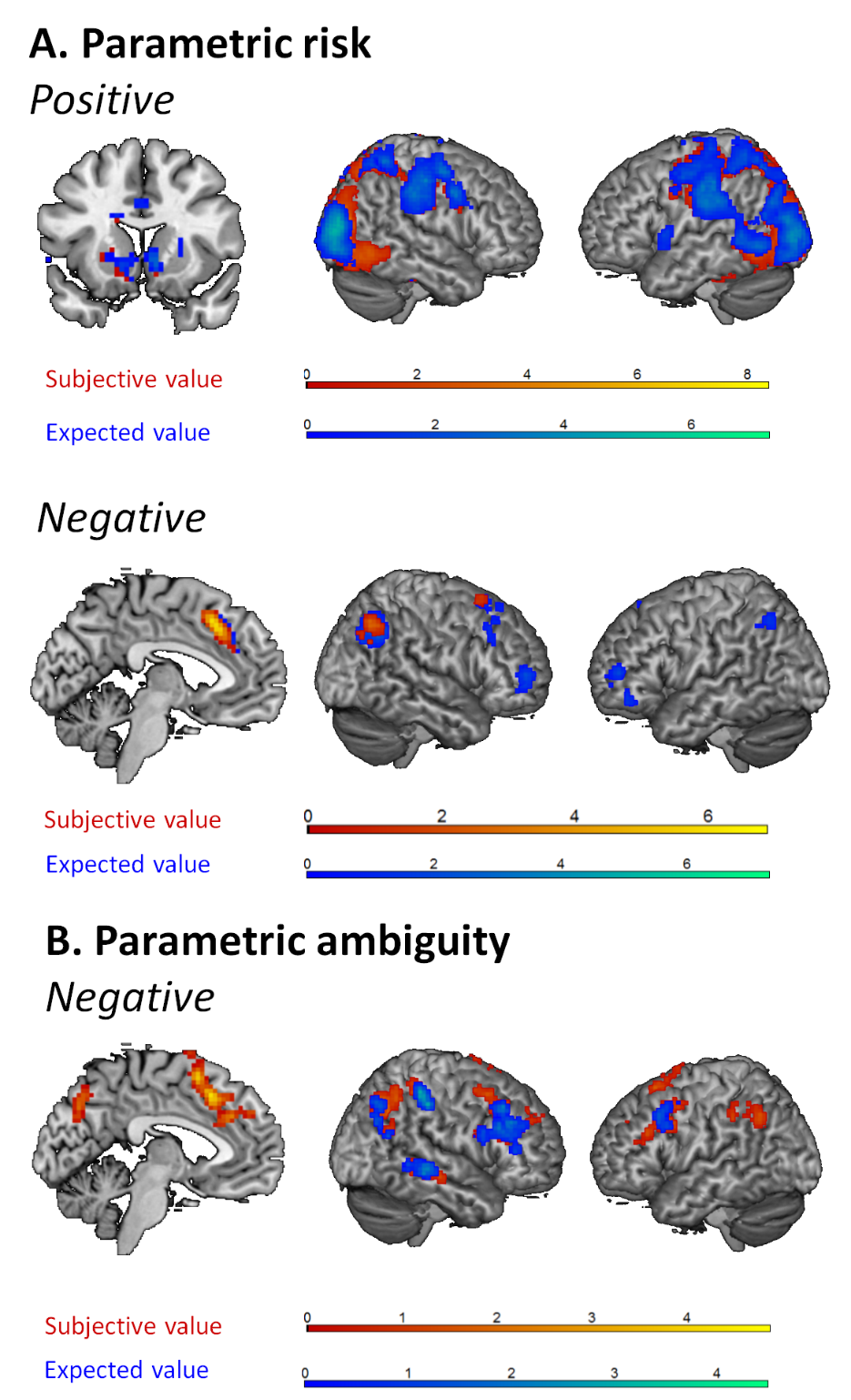


*Figure S4.* Results of the effects of **A.** risk positive (upper panel: y = 14; R; L) and risk negative (lower panel: x = -4, R; L) and **B.** ambiguity negative (x = -4; R; L). Activation in blue represents activation for expected value, activation in red represents activation for subjective value. Results were FWE cluster-corrected (*p* < .05).

| Table S1. Whole brain results for expected value. Results were FWE cluster-corrected (*p* < .05). | | | | | | | |
| --- | --- | --- | --- | --- | --- | --- | --- |
|  |  | **MNI coordinates** | | |  |  |  |
| ***Anatomical region*** | ***+/-*** | ***x*** | ***y*** | ***z*** | ***T*** | ***k*** | ***p*** |
| *EV Risk* | | | | | | | |
| R calcarine gyrus | + | 15 | -91 | 4 | 7,20 | 4750 | < .001 |
| R calcarine gyrus | + | 18 | -94 | 13 | 6,96 |  |  |
| R cuneus | + | 30 | -91 | 10 | 6,95 |  |  |
| R postcentral gyrus | + | 60 | -19 | 52 | 5,90 | 712 | < .001 |
| R caudate nucleus | + | 9 | 17 | -5 | 5,19 | 297 | < .001 |
| R superior parietal lobe | + | 27 | -52 | 64 | 5,67 | 412 | < .001 |
| R precuneus | + | 15 | -58 | 61 | 5,14 |  |  |
| R paracentral lobe | + | 12 | -31 | 52 | 4,06 |  |  |
| L insula lobe | + | -33 | -4 | 16 | 5,16 | 1124 | < .001 |
| R cerebellum | + | 33 | -40 | -29 | 4,87 | 79 | .045 |
| R cerebellum | + | 27 | -31 | -29 | 3,96 |  |  |
| R fusiform gyrus | + | 45 | -34 | -26 | 3,80 |  |  |
| L inferior frontal gyrus (pars opercularis) | + | -57 | 8 | 7 | 4,59 | 101 | .018 |
| L temporal pole | + | -57 | 8 | -2 | 3,98 | 491 | < .001 |
| L superior medial gyrus | - | -6 | 23 | 43 | 7,28 |  |  |
| R middle cingulate gyrus | - | 9 | 26 | 34 | 5,18 |  |  |
| R anterior cingulate cortex | - | 12 | 32 | 25 | 4,45 | 298 | < .001 |
| R angular gyrus | - | 48 | -58 | 40 | 5,05 | 153 | .002 |
| R middle frontal gyrus | - | 33 | 53 | 4 | 4,70 |  |  |
| R middle frontal gyrus | - | 42 | 26 | 40 | 3,90 |  |  |
| R middle orbital gyrus | - | 39 | 47 | -5 | 3,74 | 129 | .006 |
| L middle frontal gyrus | - | -33 | 50 | 10 | 4,69 |  |  |
| L middle frontal gyrus | - | -42 | 44 | -8 | 4,19 |  |  |
| L superior frontal gyrus | - | -33 | 56 | 1 | 3,60 | 98 | .02 |
| L inerior parietal lobe | - | -48 | -61 | 49 | 4,21 |  |  |
|  |  |  |  |  |  |  |  |
| *EV Ambiguity* | | | | | | | |
| R inferior parietal lobe | - | 54 | -37 | 52 | 4,48 | 120 | .008 |
| R middle temporal gyrus | - | 69 | -31 | -2 | 4,12 | 89 | .03 |
| R middle temporal gyrus | - | 60 | -46 | 1 | 3,65 |  |  |
| R middle frontal gyrus | - | 39 | 26 | 43 | 4,10 | 271 | < .001 |
| R middle frontal gyrus | - | 39 | 32 | 31 | 4,00 |  |  |
| R inferior frontal gyrus (pars triangularis) | - | 54 | 23 | 28 | 3,95 |  |  |
| R angular gyrus | - | 36 | -70 | 46 | 3,84 | 88 | .03 |
| R angular gyrus | - | 48 | -70 | 31 | 3,72 |  |  |
| R middle temporal gyrus | - | 54 | -61 | 22 | 3,63 |  |  |
| L middle frontal gyrus | - | -42 | 20 | 40 | 3,78 | 79 | .044 |
| L inferior frontal gyrus (pars opercularis) | - | -51 | 14 | 34 | 3,63 |  |  |
| L middle frontal gyrus | - | -42 | 5 | 52 | 3,25 |  |  |
| Note: EV = expected value, L = left; R = right. Anatomical labels are based on the Automated Anatomical Labeling (AAL) atlas. | | | | | | | |
